# Therapeutic Trial of Metformin and Bortezomib in a Mouse Model of Tuberous Sclerosis Complex (TSC)

**DOI:** 10.1371/journal.pone.0031900

**Published:** 2012-02-21

**Authors:** Neil Auricchio, Izabela Malinowska, Reuben Shaw, Brendan D. Manning, David J. Kwiatkowski

**Affiliations:** 1 Translational Medicine Division, Brigham and Women's Hospital, Boston, Massachusetts, United States of America; 2 The Salk Institute, San Diego, California, United States of America; 3 Department of Genetics and Complex Diseases, Harvard School of Public Health, Boston, Massachusetts, United States of America; Faculty of Pharmacy, Ain Shams University, Egypt

## Abstract

Tuberous sclerosis complex (TSC) is a human genetic disorder in which loss of either TSC1 or TSC2 leads to development of hamartoma lesions, which can progress and be life-threatening or fatal. The TSC1/TSC2 protein complex regulates the state of activation of mTORC1. *Tsc2^+/−^* mice develop renal cystadenoma lesions which grow progressively. Both bortezomib and metformin have been proposed as potential therapeutics in TSC. We examined the potential benefit of 1 month treatment with bortezomib, and 4 month treatment with metformin in *Tsc2^+/−^* mice. Results were compared to vehicle treatment and treatment with the mTORC1 inhibitor rapamycin for 1 month. We used a quantitative tumor volume measurement on stained paraffin sections to assess the effect of these drugs. The median tumor volume per kidney was decreased by 99% in mice treated with rapamycin (p = 0.0004). In contrast, the median tumor volume per kidney was not significantly reduced for either the bortezomib cohort or the metformin cohort. Biochemical studies confirmed that bortezomib and metformin had their expected pharmacodynamic effects. We conclude that neither bortezomib nor metformin has significant benefit in this native *Tsc2^+/−^* mouse model, which suggests limited benefit of these compounds in the treatment of TSC hamartomas and related lesions.

## Introduction

TSC is an autosomal dominant tumor suppressor gene syndrome in which hamartomas develop in multiple organ systems, including the heart, brain, skin, kidneys, lymphatic system, and lungs [1]. Although most lesions have a benign course, several can grow progressively necessitating clinical intervention with drug treatment and/or surgery.

TSC is due to mutations in either TSC1 or TSC2, and progressive lesions typically show complete loss of one gene product or the other [1]. The TSC1/TSC2 protein complex serves a unique function in negatively regulating the amount of GTP-RHEB in the cell, by acting as a GTPase activating protein (GAP) for RHEB [2], [3]. GTP-RHEB is an essential activator of mTOR complex 1 (mTORC1), with downstream effects on transcription and translation, cellular metabolism, autophagy, ribosome biogenesis, cell size control, and cell proliferation [4], [5]. mTORC1 is constitutively activated in cells lacking either TSC1 or TSC2 and in hamartomas from TSC patients [6].

Rapamycin (sirolimus) and related drugs (everolimus), which bind to and inhibit mTORC1 through FKBP12, have shown clinical activity for treatment of several manifestations of TSC, including renal angiomyolipomas, pulmonary lymphangioleiomyomatosis, and brain subependymal giant cell astrocytomas [7]–[10]. However, rapamycin does not cause complete regression of disease in most instances, and cessation of treatment can lead to regrowth of hamartoma lesions [8]. Rapamycin is also an immunosuppressant, and although it is well tolerated in most patients during follow-up of up to 5 years, there is concern that long-term treatment may lead to significant cumulative or otherwise unexpected toxicity. A fraction of patients also do not tolerate the drug, mainly due to oral mucositis [7]–[10].

Other therapeutic approaches to control the growth of TSC-related tumors have been suggested. Loss of the TSC protein complex and activation of mTORC1 leads to elevated and uncontrolled protein synthesis, which leads to endoplasmic reticulum (ER) stress and induction of the unfolded protein response (UPR) [11]. One potential therapeutic approach therefore is to exacerbate ER stress in tumor cells lacking the TSC genes by treatment with proteasome inhibitors such as bortezomib [12]. Bortezomib is approved for clinical use in multiple myeloma [13], and is thought to cause the selective death of myeloma cells through induction of apoptosis. A second alternative therapeutic approach is the use of AMPK activators. AMPK inhibits the activation of mTORC1 through both activating phosphorylation of TSC2 [14], [15], and inhibitory phosphorylation of mTORC1 on the core component Raptor [16]. In addition, compounds which induce ATP depletion and energy stress can also selectively kill cells lacking TSC1 or TSC2 [17]. Several AMPK inhibitors are available for clinical investigation. Metformin is a biguanide compound which activates AMPK by elevating cellular AMP levels [18], and is recommended as first line therapy for adult onset diabetes mellitus, to reduce serum glucose and Hemoglobin A1c levels [19]. It is generally well tolerated and has been taken by some patients for more than a decade. In addition, metformin has been shown to prevent tumorigenesis in mouse model studies [20], [21].

*Tsc2^+/−^* mice develop renal cystadenomas that progressively develop as the mouse ages, with increasing cellular atypia in some lesions that progress to renal cell carcinoma [22]. The *Tsc2^+/−^* renal cystadenoma phenotype is most pronounced in the A/J strain of mice (unpublished data). Here we used *Tsc2^+/−^* A/J mice to examine the potential benefit of bortezomib and metformin treatment for renal cystadenomas, in comparison to rapamycin and vehicle controls.

## Results

Cohorts of A/J strain *Tsc2^+/−^* mice were treated with one of 5 regimens: 1) rapamycin given at 6 mg/kg IP three days per week (n = 9); 2) vehicle given on the same schedule (n = 5); 3) bortezomib given at 0.8 mg/kg SC two days per week (n = 8); 4) metformin given in 5% sucrose drinking water at 300 mg/kg per day (n = 10); and 5) 5% sucrose drinking water (n = 8). Cohorts were chosen to be balanced by sex; the first three cohorts were treated concurrently, as were the last two cohorts. Since rapamcyin has previously been shown to cause a marked reduction in tumor volume in *Tsc2^+/−^* mice, it was given for one month only, as we have done previously [23]. Bortezomib was also given for one month only, as it may be considered a relatively active therapeutic with potential toxicity and side-effects, such that long-term administration in patients would not be desirable. The dosage used has been established as an MTD in previous studies in xenograft models, and is effective for sensitive tumors [12]. In contrast, since metformin is taken by many patients for many years, it was given to these mice for a period of 4 months, modeling a long-term preventive mode of treatment. The dosage of metformin was taken from a previous study in which this dose was effective in prevention of tumors in a combined LKB1-PTEN mouse model [21]. All mice were sacrificed at 5 months of age. Mice received rapamycin and bortezomib from age 4 to 5 months; and received metformin from age 1 month to age 5 months. Note that one mouse treated with bortezomib died unexpectedly during treatment. Otherwise there was no apparent toxicity or difference in weight among any of the cohorts (Table 1).

**Table 1:** Body weight of mice on treatment.

Treatment	Age	Average weight	Standard deviation	Median	Number of mice
IP vehicle	4 m	26.2	1.91	25.5	5
IP vehicle	5 m	25.2	1.91	24.3	5
Rapamycin	4 m	25.2	1.38	25.0	9
Rapamycin	5 m	25.1	1.15	25.3	9
Bortezomib	4 m	24.8	1.51	24.6	7
Bortezomib	5 m	23.4	1.59	23.3	7
Sucrose	1 m	18.3	2.28	18.5	8
Sucrose	2 m	21.3	1.95	21.3	8
Sucrose	3 m	23.7	1.66	23.7	8
Sucrose	4 m	25.6	2.16	25.6	8
Sucrose	5 m	25.2	2.61	25.2	8
Metformin	1 m	16.7	1.10	16.8	10
Metformin	2 m	21.4	2.59	21.2	10
Metformin	3 m	23.9	3.41	23.3	10
Metformin	4 m	25.1	2.37	24.7	10
Metformin	5 m	26.3	2.47	26.3	10

**Figure 1:**
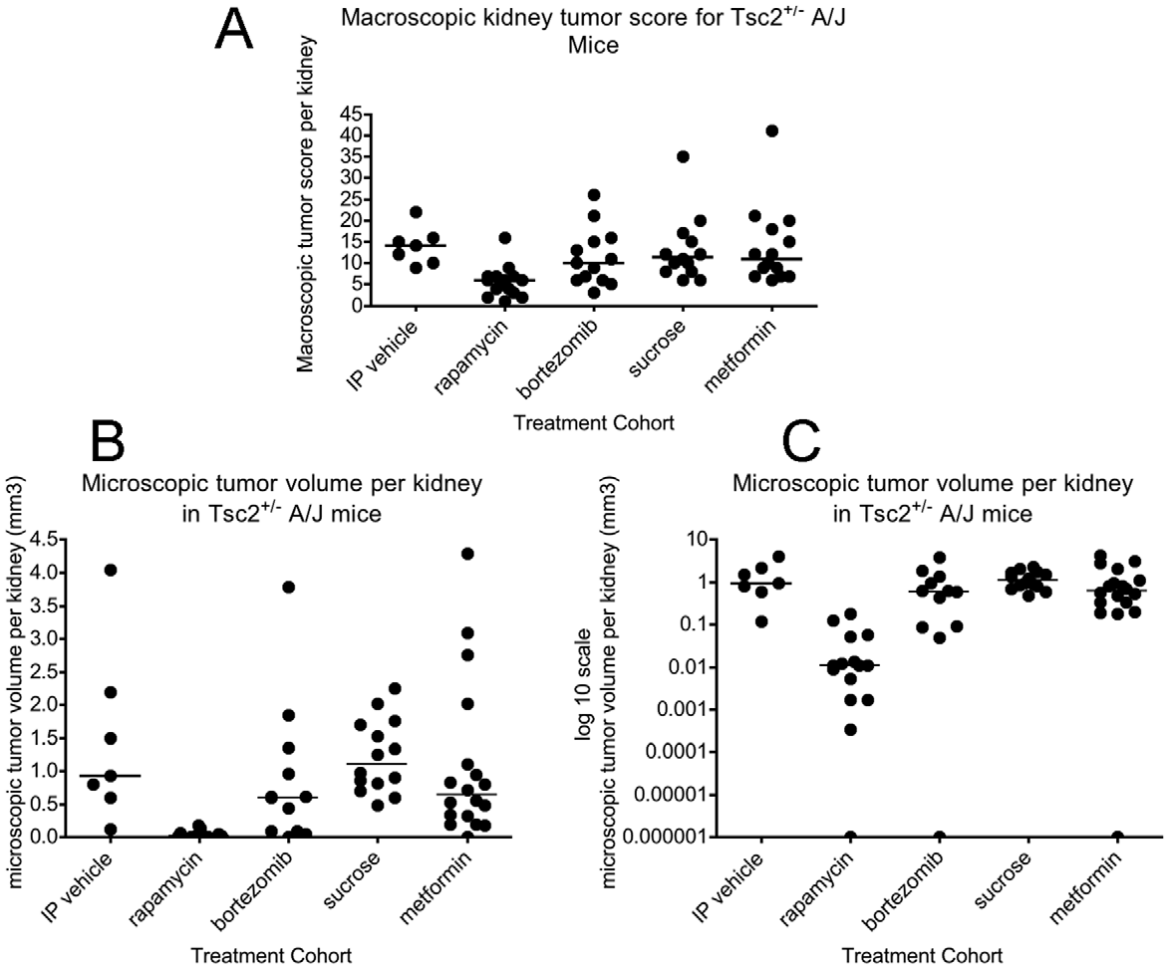
Macroscopic and microscopic kidney tumor scores for *Tsc2^+/−^* mice treated with five different treatment regimens. Scores are shown for each kidney available from each mouse in these cohorts. A. Macroscopic kidney tumor scores. B–C. Microscopic kidney tumor volume is shown twice, on a linear y axis scale (B) and on a logarithmic y axis scale (C). For B and C, the number of mice and kidneys examined were: 5 and 7, 9 and 15, 7 and 12, 8 and 14, and 10 and 18, respectively, for IP vehicle, rapamycin, bortezomib, sucrose, and metformin cohorts, respectively.

**Figure 2:**
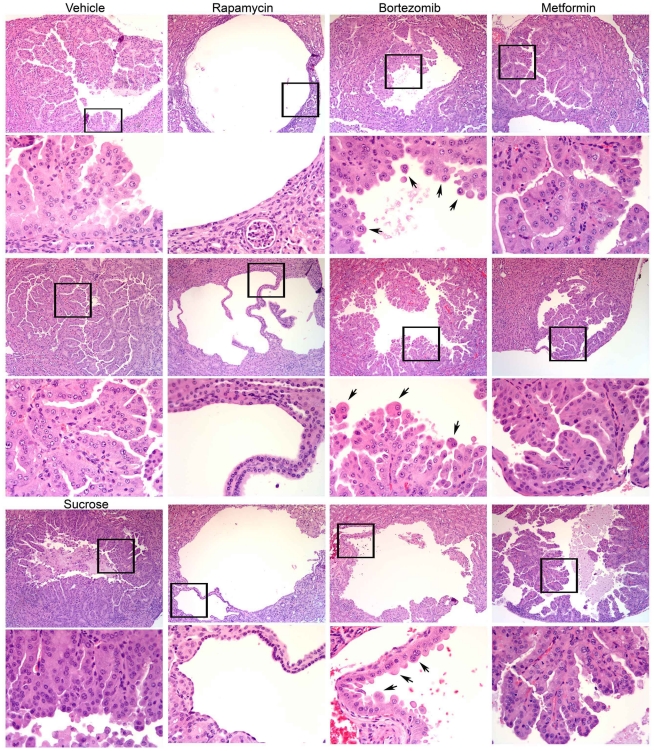
Renal cystadenoma histology in the treated mice. Representative tumor images are shown for each treatment cohort, selected from the kidneys with the largest tumor volume for each. Three cystadenoma each are shown at 100×, with a portion of the tumor indicated by the frame shown at 400× (below). Cystadenomas from control mice are shown in the first column, two from IP vehicle treated mice, and one from a sucrose treated mouse. Note the cystic nature of the tumors from the rapamycin-treated mice, as well as the flattened, thin nature of the cyst-lining cells, in contrast to cystadenomas from all other mice. Arrows point to prominent enlarged but viable cells in the bortezomib-treated mice.

Among the five treatment cohorts, the only cohort showing a significant difference in tumor extent as assessed by gross observation was the rapamycin treatment cohort (Figure 1A). Gross tumor extent was significantly reduced in the rapamycin cohort in comparison with IP vehicle (p = 0.0012, Mann Whitney test), as well as in pairwise comparison with every other treatment cohort (p≤0.01). All comparisons among other treatment cohorts were not significant (p>0.2 for each other pairwise comparison). Similarly, quantitative scoring of microscopic tumor volume per kidney showed that the rapamycin-treated mice had a markedly reduced tumor volume in comparison with every other treatment group, with p = 0.0004 in comparison to the IP vehicle cohort, and p≤0.001 in comparison to the other 3 cohorts (Figure 1B). Indeed, visualization of tumor volume on a log scale (Figure 1C) illuminated the ∼100-fold difference in median tumor volume in the rapamycin-treated cohort in comparison to all other cohorts (median tumor volume 0.93 mm2 for IP vehicle, and 0.011 mm2 for rapamycin). This figure also illustrates the marked variability in the extent of tumor formation in this pure strain genetic model, likely reflecting the important effect of stochastic variation in second hit genetic events important in tumor development. In contrast, by quantitative microscopic assessment, there were only marginal differences among the four other cohorts. There was a trend towards a difference in comparison of both the bortezomib cohort and the metformin cohort with the sucrose cohort, p = 0.054 and 0.084 respectively. However, comparison with the IP vehicle group showed no significant difference, p≥0.16. Combining the IP vehicle and sucrose cohorts, and repeating the comparisons gave p values of 0.041 and 0.083 for the comparisons of the bortezomib and metformin cohorts with the combined vehicle cohort respectively. Microscopic review of renal cystadenoma histology demonstrated that there was no apparent change in the appearance of tumors from the bortezomib and metformin cohorts in comparison to controls (Figure 2). In contrast, the rapamycin-treated mouse tumors were almost entirely cystic, and the cyst-lining cells had a flattened appearance in comparison to tumor cells from the other four cohorts (Figure 2), as has been noted previously in rapamycin or RAD001 treated *Tsc2^+/−^* mice [23].

**Figure 3:**
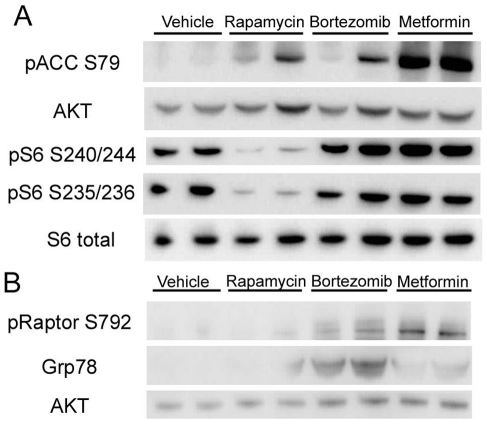
Immunoblot analysis of kidney lysates to examine therapeutic specificity. *Tsc2^+−^* A/J strain mice were treated with vehicle, rapamycin, bortezomib or metformin for 1 week, and kidney lysates were prepared for immunoblot analysis. A) Blot strips were incubated with the antibodies against pACC-S79, AKT, pS6-S235/236, pS6-S240/244 and S6. B) Blot strips incubated with antibodies against pRaptor-S792, GRP78 and AKT.

**Figure 4:**
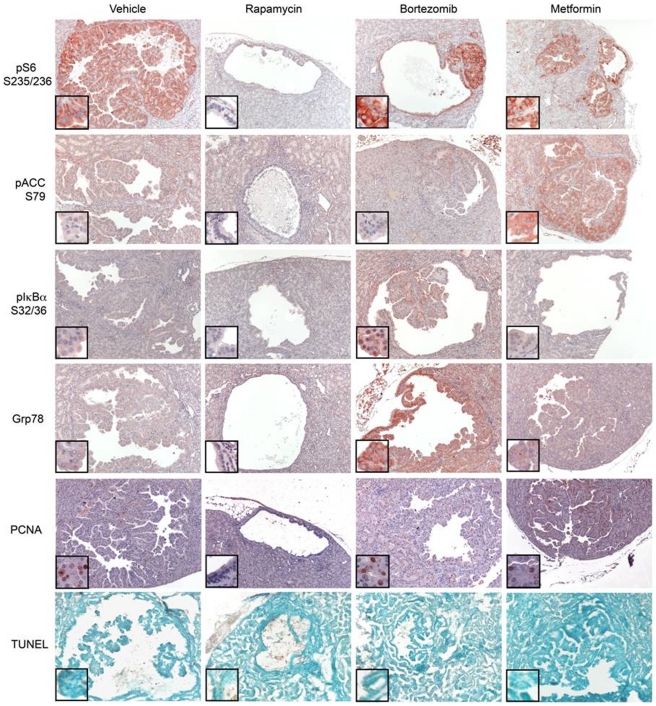
Immunohistochemistry (IHC) analysis of therapeutic effects. *Tsc2^+/−^* A/J strain mice of ages 9–10 months were treated with drugs for 1 week, and kidneys prepared for histology. Sections were prepared and stained using pS6-S235/236 (red), pACC-S79 (red), pIκBα-S32/34 (red) and GRP78 (red) antibodies. The bottom panel shows apoptosis assessed by TUNEL method. Representative sections are shown. All images shown are taken at 100× magnification. Insets show portions of the tumor at higher magnification (400×).

To confirm that these drugs were hitting their intended molecular targets in the kidneys of these mice, two studies were performed. Separate sets of *Tsc2^+−^* AJ strain mice were treated with the drugs for one week, and sacrificed for immunoblot analysis of kidney tissue lysates. Antibodies used in this assay were designed to examine the activity of mTORC1 (phospho-S6-S235-236, pS6-S235; pS6-S240/S244) [6]; the activity of AMPK (phospho-ACC-S79 and pRaptor-S792) [24]; and proteasome inhibition (GRP78/BiP, phospho-IκBα-S32/36) [25]. Levels of pS6-S235/S236 and pS6-S240/S244 were markedly reduced in kidney lysates from mice treated with rapamycin, and were not changed in mice treated with the other agents (Figure 3A). Similarly, mice treated with metformin showed an increase in both pACC-S79 (Figure 3A) and pRaptor-S792 (Figure 3B) levels. Bortezomib treatment markedly induced GRP78, consistent with this drug acting as a potent activator of the unfolded protein response (UPR) (Figure 3B). Bortezomib treatment also led to an increase in pRaptor-S792 levels, though to a smaller extent than metformin treatment (Figure 3B). pACC-S72 levels were somewhat variable in rapamycin- and bortezomib-treated mice, but were much less than what was seen with metformin treatment (Figure 3A). Immunohistochemistry analysis performed on tumors from *Tsc2^+/−^* mice treated with these agents for one week confirmed these findings (Figure 4). There was a complete absence of pS6-S235/236 staining in rapamycin-treated mouse tumors, in contrast to mice treated with the other drugs. Metformin led to an increase in pACC-S79 levels. Bortezomib led to an increase in both pIκBα-S32/36 and GRP78 levels. Apoptosis was not appreciated in these mouse tumors after one week of any treatment by TUNEL staining (Figure 4).

## Discussion

In this study, we have confirmed the dramatic therapeutic effectiveness of rapamycin treatment of *Tsc2^+/−^* mice for the renal cystadenoma lesions which are known to develop through a 2-hit mechanism of complete loss of Tsc2 expression [22]. Multiple previous studies have demonstrated this response to rapamycin and its analogs [23], [26], [27]. However, it was important to perform this positive control concurrently with our test compounds.

In contrast, we saw little or no therapeutic benefit in *Tsc2^+/−^* mice in response to treatment with bortezomib. There was marginal evidence of benefit when comparing the bortezomib cohort to the combined IP vehicle and sucrose control cohorts (p = 0.041). This agent is currently FDA-approved for treatment of multiple myeloma [13], and acts by inhibiting the proteasome to enhance ER stress in malignant plasma cells that produce large amounts of immunoglobulin, leading to inhibition of proliferation and apoptotic cell death [28]. This effect was not seen in the mice treated in our study, as indeed, there was no appreciation of cell size increase in mice treated for either one week or one month of bortezomib (Figures 2 and 4). However, our studies show that the UPR pathway was enhanced in the normal tissues and tumor lesions in these mice, as manifest by increased levels of GRP78/BiP, a known target of ATF6 [29]. Therefore, it appears that the level of increase in ER stress and UPR was insufficient to induce cell death in this particular Tsc2+− genetic model.

Similarly, metformin also failed to demonstrate therapeutic benefit in this model. Mice were treated with a conventional, relatively high dose of drug [21], and our analyses indicated that there was induction of AMPK activation in response to treatment (Figures 3 and 4). Metformin was chosen for use in this study because of its widespread use in the treatment of diabetes mellitus [19], and lack of toxicity or major side-effects when taken for years at a time, important considerations for the concept of long-term preventive treatment. As noted earlier, metformin activation of AMPK leads to phosphorylation and activation of TSC2 [14], [15]. AMPK also phosphorylates Raptor to attenuate mTORC1 activity [16]. Metformin treatment has been used to reduce tumor severity in both *Lkb1^fl/+^ Pten^+/−^* mice [21], and in tobacco carcinogen-induced lung tumorigenesis in A/J strain of mice, the same strain used here [20]. However, it is notable that the reduction in tumor severity was modest through statistically significant in both of these studies, and IP administration at a high dose was required to achieve the greatest benefit in the lung cancer model [20]. We suspect that the complete absence of Tsc2 in these *Tsc2^+/−^* cystadenoma lesions leads to strong activation of mTORC1 due to constitutive high levels of RHEB-GTP, which makes these lesions resistant to the lesser effect of Raptor phosphorylation. This is reflected in a lack of decrease in mTORC1 signaling in the lesions following metformin treatment, with persistent strong pS6-S235/236 expression, in contrast to the effects seen with rapamycin treatment (Figure 3, 4). However, it is notable that part of the therapeutic benefit of metformin in previous tumor studies may relate to effects on circulating insulin and insulin-like growth factor levels, as well as energy stress [20], [30].

The current studies are important in indicating the likely limited therapeutic benefit of both bortezomib and metformin for treatment of TSC and related disorders, including renal angiomyolipoma and pulmonary lymphangioleiomyomatosis (LAM). It is possible that a more potent AMPK activator such as AICAR or phenformin would be more effective at inhibition of both mTORC1 signaling and growth in *Tsc2^+/−^* cystadenoma lesions since they are also more effective in *in vitro* studies on TSC2-deficient fibroblasts [16]. However, those agents are not readily available for clinical testing in the US population. Indeed, development of more potent bioavailable AMPK activators for clinical use would be very helpful for further analysis of this potential therapeutic approach. Although there is a continuing need for therapeutic development and refinement for treatment of TSC and related disorders, our data suggests that metformin and bortezomib will not have major benefit.

## Materials and Methods

### Ethics statement

All procedures were carried out in accordance with the Guide for the Humane Use and Care of Laboratory Animals, and the study was approved by the Animal Care and Use Committee of Children's Hospital, Boston.

### Mouse procedures

*Tsc2^+/−^* mice, originally generated in this laboratory [22], were serially crossed with A/J mice for over 5 generations. These pure strain mice were used in all experiments.

### Standard histology and tumor assessment

Mouse kidneys were removed rapidly after euthanasia, and were fixed overnight in 10% formalin. Gross scoring of kidney tumor lesions was performed by a single observer (NA). The gross tumor score for each kidney was determined as a summed score for all lesions in a kidney, scoring each individual tumor grossly as follows: 1 for tumors <1 mm; 2 for 1 to 1.5 mm; 5 for 1.5 to 2 mm; 10 for >2 mm [23]. Kidneys were then prepared for histologic evaluation in stereotypical fashion by cutting the kidney into sections at 1 mm intervals throughout its length.

Microscopic kidney tumor scores were determined in a semi-quantitative fashion by a single blinded observer (IM). The set of 1 mm interval sections were prepared as H&E-stained 8 micrometer sections. Each tumor or cyst identified was measured to determine its length and width in two dimensions, as well as the percent of the lumen filled by tumor (this was 0% for a simple cyst, and 100% for a completely filled, solid adenoma). These measurements were converted into a measurement of tumor volume per lesion using the following formula. Tumor volume = maximum(tumor percent, 5)/100 * 3.14159/6 * 1.64 * (tumor length * tumor width)**1.5 [31]. The total tumor volume per kidney was then equal to sum of the tumor volume of each lesion identified. Comparisons between sets of mice for tumor measurements were made using the non-parametric Mann Whitney test in Prism (GraphPad Software, Inc., v4.0a).

### Immunoblotting

Mouse kidneys were harvested for immunoblotting by rapid post-mortem freezing in liquid nitrogen and were stored at −80°C until further use. Lysates from kidney samples were prepared by standard methods [23]. Protein concentrations were determined using the Bradford Assay, and equal amounts were separated by electrophoresis on pre-cast 4–12% Bis-Tris gels (Invitrogen) and transferred onto PVDF membranes. The membranes were blocked with 5% nonfat dry milk in TBS-T (Tris Buffered Saline-10% Triton-X) for one hour at room temperature. Primary antibodies were diluted in 5% bovine standard albumin (BSA) in PBS, 0.1% Tween20 (pH 7.4) solution and were applied to the membranes for overnight incubation at 4°C in a wet chamber. Antibodies used for Western blotting were as follows: pACC (S79, Millipore,Temecula, CA); Akt (Santa Cruz Biotechnology, Santa Cruz, CA); GRP78/BiP (Enzo, Farmingdale, NY; formerly Stressgen); and pIκBα (S32/36), pS6 (S240/244), pS6 (S235/236), total S6 and pRaptor (S792) (all from Cell Signaling Technology, Bedford, MA).

After extensive washing, HRP-conjugated secondary antibodies (Santa Cruz) were applied for 1 hour at room temperature. SuperSignal West Pico (ThermoScientific) chemiluminescence reagents were used to detect antibody binding. Images were collected digitally using the Syngene G-box iChemi XT GeneSnap Program (Version 7.09.06).

### Immunohistochemistry

Eight micrometer paraffin sections were deparaffinized with a xylene and alcohol series, treated with Target Retrieval Solution pH 6.1 (Dako, Carpinteria, CA), blocked with 3% H2O2 in methanol, and then put in 5% normal goat serum in 0.1% Triton X in PBS. Sections were incubated with primary antibodies overnight at 4°C, washed, and incubated with secondary antibody conjugated with horseradish peroxidase (HRP). AEC (3-amino-9-ethylcarbazole, Envision+System Dako) was then applied to generate a color reaction. Slides were then counterstained with hematoxylin (Dako). Antibodies used for staining were: pACC-S79 (1∶100, Millipore #07-303); anti-pIκBα-S32/36 (1∶100, Cell Signaling, #9246); anti-pS6-S235/236 (1∶1000, Cell Signaling, #2211), GRP78 BiP (1∶3000, Abcam, #21685).Apoptosis was assessed on frozen kidney sections (20 µm-thick) with ApopTag Peroxidase In Situ Apoptosis Detection Kit (Millipore, formerly Chemicon) by the TUNEL method and counterstained with methylene green (Sigma).

### Drug handling and administration

Metformin was purchased from Sigma-Aldrich (St. Louis, MO; D15,095-9), and was dissolved in 5% sucrose and provided to the mice in their drinking water, such that the mice received 300 mg/kg daily. Mice drank 3–15 ml/day, and the concentration was adjusted every other day according to their consumption and body weight, to achieve the desired daily dose. A sucrose control group of mice received 5% sucrose as drinking water without additive. Both the metformin and the sucrose controls received treatment for 4 months, from age 1 month to age 5 months.

Rapamycin was purchased from LC laboratories (Woburn, MA). A 20 mg/ml stock was made using ethanol, and mixed daily for injection with sterile vehicle (0.25% PEG-200, 0.25% Tween-80). Mice were treated with rapamycin by intraperitoneal (IP) injection at 6 mg/kg three times per week for one month. Control mice received the vehicle solution IP on the same schedule. Bortezomib was also purchased from LC laboratories. A 25 mg/ml stock was made using DMSO or PBS, and was diluted daily with sterile PBS. Mice were treated with bortezomib at 0.8 mg/kg subcutaneously two times per week (three days apart) for one month [12].

For short term treatment studies, 2 *Tsc2^+−^* AJ strain mice of age 9–10 months each were treated with rapamycin, bortezomib, metformin, or sucrose water in the same doses as above for a period of one week only. They were then sacrificed for rapid collection of kidneys; one half of one kidney was rapidly frozen for immunoblot analysis, the other half lightly fixed and prepared in 30% sucrose for frozen sections for Apoptag analysis. The other kidney was fixed and used for immunohistorchemistry.
